# Molar Hypomineralisation: A Call to Arms for Enamel Researchers

**DOI:** 10.3389/fphys.2017.00546

**Published:** 2017-08-03

**Authors:** Michael J. Hubbard, Jonathan E. Mangum, Vidal A. Perez, Garry J. Nervo, Roger K. Hall

**Affiliations:** ^1^Department of Paediatrics, University of Melbourne Melbourne, VIC, Australia; ^2^Department of Pharmacology and Therapeutics, University of Melbourne Melbourne, VIC, Australia; ^3^Department of Pediatric Stomatology, University of Talca Talca, Chile

**Keywords:** enamel defects, enamel opacities, dental caries, amelogenesis imperfecta, dental fluorosis, enamel hypoplasia, translational research, networked research

## Abstract

Developmental dental defects (DDDs, hereafter “D3s”) hold significance for scientists and practitioners from both medicine and dentistry. Although, attention has classically dwelt on three other D3s (amelogenesis imperfecta, dental fluorosis, and enamel hypoplasia), dental interest has recently swung toward Molar Hypomineralisation (MH), a prevalent condition characterised by well-delineated (“demarcated”) opacities in enamel. MH imposes a significant burden on global health and has potential to become medically preventable, being linked to infantile illness. Yet even in medico-dental research communities there is only narrow awareness of this childhood problem and its link to tooth decay, and of allied research opportunities. Major knowledge gaps exist at population, case and tooth levels and salient information from enamel researchers has sometimes been omitted from clinically-oriented conclusions. From our perspective, a cross-sector translational approach is required to address these complex inadequacies effectively, with the ultimate aim of prevention. Drawing on experience with a translational research network spanning Australia and New Zealand (The D3 Group; *www.thed3group.org*), we firstly depict MH as a silent public health problem that is generally more concerning than the three classical D3s. Second, we argue that diverse research inputs are needed to undertake a multi-faceted attack on this problem, and outline demarcated opacities as the central research target. Third, we suggest that, given past victories studying other dental conditions, enamel researchers stand to make crucial contributions to the understanding and prevention of MH. Finally, to focus geographically diverse research interests onto this nascent field, further internationalisation of The D3 Group is warranted.

Developmental dental defects (DDDs), hereafter termed D3s per recent translational usage^1^, hold significance for scientists and practitioners from both medicine and dentistry. Dentally, D3s are commonly associated with increased risk for toothache, decay (caries) and concerns about appearance, each of which may require non-standard approaches to management. Medically, although childhood illness clearly underlies many D3s, the lack of specific causalities and prognostics precludes doctors from taking preventive measures. And scientifically, despite evidence that many D3s involve disruption of the enamel-forming cells (ameloblasts), today's scant pathomechanistic understanding carries little in the way of clinical value. These knowledge gaps hold importance not only for naturally-formed teeth, but also for future aspirations to bioengineer replacement teeth free of D3s. Classically, the collective attention of dentists, doctors, and scientists has dwelt mostly on three types of D3 that affect enamel, namely amelogenesis imperfecta, dental fluorosis, and enamel hypoplasia. Multiple victories have arisen from these research efforts which have spanned more than a century (see Box 1). Over the past decade however, dental attention has swung toward a condition primarily affecting back teeth and which we term Molar Hypomineralisation (or “chalky molars” in public settings^2^).

Box 1 **Past victories involving enamel research**

**“Tetracycline-Stained Teeth”**

From the mid-1950s, many children were found to have abnormally coloured teeth at the time of eruption. Such “enamel staining” generally worsened with age, progressing from yellow to grey/brown. Researchers soon linked this problem to paediatric use of tetracycline, a newly available antibiotic. This side-effect, which also affected dentine and bone, was exploited scientifically to elucidate the developmental timing of enamel formation and allied defects. With few exceptions, tetracycline-stained teeth are now consigned to history thanks to evidence-based interventions at multiple levels (medical, pharmacy, industry, government).

**Dental Fluorosis (“Mottled Enamel”)**

In the 1920s, research across the USA led to evidence that populations exposed to low amounts of fluoride in drinking water had lesser amounts of dental decay. Higher fluoride exposures were linked to “fluorosis” (i.e., enamel with defects ranging from white flecks through to brown pits). A century of research has since delivered comprehensive understanding of the effects of fluoride on populations, individuals, teeth, cells, and minerals. Enamel has been at the heart of this research. Today's evidence-base enables fluoride to be used safely and effectively in a variety of dental, medical and public-health settings. A global decrease in tooth decay has occurred consequently.

**Tooth Decay (Dental Caries)**

By the 1960s and with rates of tooth decay plummeting, attention turned to the earliest stages of dental caries in superficial enamel (i.e., so-called “white-spot lesions”) with the aim of preventing or reversing the disease process. Researchers elucidated how enamel is attacked by acids from dental plaque and established various procedures for reversing or arresting such damage. These developments have led to a new generation of dentistry whereby surgical “drilling and filling” (dental restorations) may be preempted by various preventive and non-surgical early interventions.

**Amelogenesis Imperfecta (“AI”)**

Early in the twentieth century it was recognised that some types of enamel defect were inheritable, leading to recognition of a complex genetic disorder termed AI (meaning “imperfect enamel formation”). Recently researchers have identified many of the genes involved and aligned these with different types of enamel abnormality. AI enamel has been characterised in animal models and humans. Genetic testing is now becoming available for some types of AI and improved dental treatments have arisen from understanding the peculiarities of AI enamel.

**Enamel Hypoplasia (“Pits and Grooves”)**

By the late nineteenth century, enamel hypoplasias (meaning “thin enamel”) were linked with childhood illness, poor appearance, and increased risk of decay. Researchers found that hypoplasia has many causes and occurs at an earlier stage of enamel formation than hypomineralisation. Thanks to better paediatric health today, enamel hypoplasias are generally less prevalent but remain of concern in some situations (e.g., high-caries-risk, vitamin D insufficiency).

## Molar hypomineralisation, a silent public health problem

Molar Hypomineralisation (MH) is emerging as a costly, yet largely silent, challenge to public health. Several key aspects can be identified, as summarised in the first column of Table 1. Foremost, the prevalence of MH is disturbingly high, with the commonest variant affecting 1-in-6 children worldwide (i.e., 17% averaged from 59 studies)^3^. In this presentation which is commonly termed “Molar-Incisor Hypomineralisation,” one or more of the permanent first-molars (“6-year molars”) are affected, sometimes with co-involvement of contemporaneous front teeth (i.e., “adult” incisors and canines). Deciduous second-molars (“baby” or “2-year molars”) are also commonly afflicted, perhaps half as frequently as 6-year molars, and some children suffer both presentations in succession. A diagnostic hallmark of MH, that distinguishes it from amelogenesis imperfecta and classical chronological D3s including fluorosis, is the sporadic occurrence of distinctively well-delineated hypomineralised enamel lesions (termed “demarcated opacities”) on anywhere between one and all-four teeth of each type^4^. With molars the main victim, MH brings considerable risk for dental breakdown and caries. Such carious lesions often manifest atypically, both in their unusually rapid progression through abnormal enamel and by being located in areas usually devoid of dental plaque (e.g., cusps and upper smooth surfaces). Toothache (dental pain) is another frequent manifestation of MH and often causes sufficient discomfort to trigger oral hygiene avoidance, exacerbating caries risk. Unsurprisingly, these fundamentally weakened teeth introduce treatment challenges for dentist and patient alike, including difficulty obtaining local anaesthesia, higher failure rates for restorations, and premature extractions with allied orthodontic need^5^. So for individual sufferers and their families, MH often inflicts significant longterm costs in financial and quality-of-life terms (e.g., dental costs, toothache, disrupted bite, and poor appearance). At population level, MH implicitly poses a substantial socioeconomic burden given its high prevalence and overlap with conventional risk factors for childhood caries (Casamassimo et al., 2009), but these costs remain to be properly quantified^6^. Regards causation, MH has been broadly linked with illnesses at the time developing enamel is being hardened (i.e., infancy in the case of 6-year molars), but specific or multifactorial causes have yet to be proven. Indeed, it still remains to be determined whether such causes relate to childhood diseases *per-se*, or instead to therapeutics commonly used in their treatment or environmental toxins. These uncertainties aside however, it does seem reasonable to anticipate that, once these research questions are answered, MH may well become largely preventable through medical or public-health interventions (Mangum et al., 2010).

**Table 1:** Comparing molar hypomineralisation with other major D3s.

	**Molar hypomin (MH)**	**Dental fluorosis**	**Enamel hypoplasia**	**Amelogenesis imperfecta**
Child prevalence (permanent teeth)	1-in-6 (≈17%)^a^	1-in-10 (≈10%)^b^	1-in-20 (≈5%)^c^	1-in-10,000 (≈0.01%)^d^
Commonest teeth affected	6-year molars^e^	Any tooth	Any tooth	All teeth
How many teeth affected	1 to all 4 of each tooth type	All front teeth often	Several teeth often	Front and back teeth
Adds risk for	Toothache, decay, cosmetic issues	Cosmetic issues mostly	Decay, cosmetic issues	Toothache, decay, cosmetic issues
Dental consequences	Fillings, extractions, orthodontic need	Cosmetic dentistry	Cosmetic dentistry, fillings	Fillings, extractions, tooth replacement
Cost to family	Medium^f^	Low	Low	High
Costs to society	High^g^	Low	Low	Low
Cause	Infantile illness	Fluoride excess	Infantile illness	Genetic mutation
Preventable?	Potentially	Yes	Largely	No

a Average value from 59 prevalence studies worldwide; www.thed3group.org/prevalence.html

b *Prevalence of dental fluorosis is often lower and sometimes much higher than 10% (e.g., due to dietary habits in some communities or exposure to naturally high levels of fluoride in drinking water)*.

c *Prevalence of enamel hypoplasia ranges from 1% in developed countries (e.g., 0.7% in New Zealand; Mahoney and Morrison, 2011) to much higher levels in communities with suboptimal paediatric healthcare [e.g., 11% in Brazil (Lunardelli and Peres, 2005) and 99% in Australian aboriginals (Pascoe and Seow, 1994)]. Accordingly an arbitrary value of 5% is used here*.

d *Global prevalence of amelogenesis imperfecta remains poorly characterised, but a range from 1-in-700 to 1-in-14,000 is often cited (Sneller et al., 2014)*.

e *Permanent first molars (“6-year molars”) are affected most commonly, followed by incisors, second molars (“12-year” molars) and primary second molars (“2-year” molars). All other teeth in the primary and permanent dentitions are affected more rarely*.

f *Lacking normal hardness, MH teeth are often difficult to treat and so restorations frequently don't last as long as usual. Extractions are common and can lead to costly orthodontic needs*.

g www.thed3group.org/economic-cost.html

**Figure 1:**
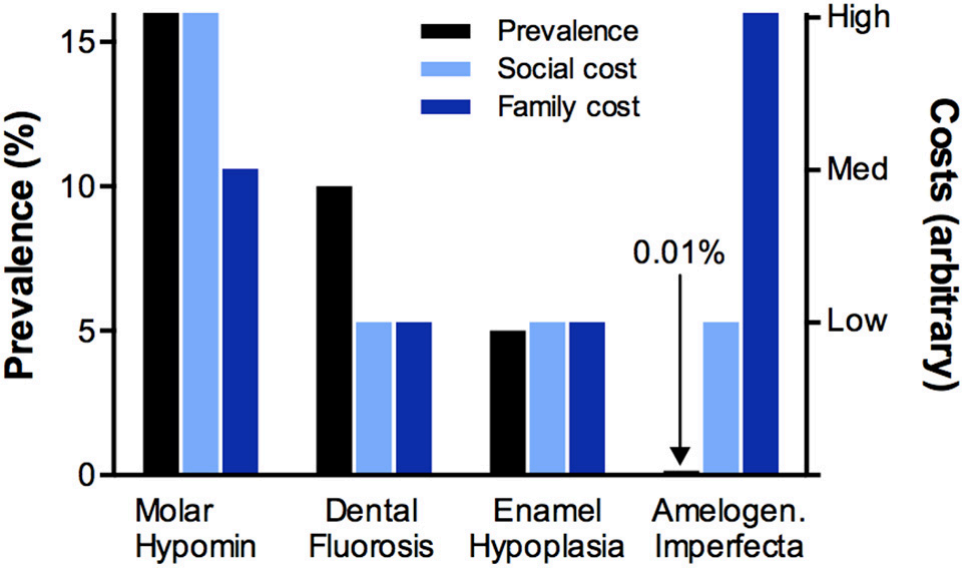
Relative impacts of the four major D3s. Prevalence, and (arbitrary) family costs and social costs were taken from Table 1 and represented comparatively in different colours as indicated. It is apparent that MH is the commonest D3, the most costly to society, and often burdensome for families (in terms of suffering, time and money). In contrast, although amelogenesis imperfecta can be quite devasting for the families concerned, this genetic disorder is relatively rare and so poses a low burden for society. Dental fluorosis and enamel hypoplasia also have lesser impacts than MH under the general population settings of Table 1.

*To strengthen comparison, a typical version of each disorder as seen in the recently-erupted permanent teeth of healthy schoolchildren was used. Representative prevalence values are given for developed countries, realising that significant differences exist in some other parts of the world. The family and social costs were ranked arbitrarily (as high, medium, low), taking into account prevalence, health risks and presumptive economic cost. High-impact features are shaded (orange) and reportrayed comparatively in Figure 1*.

When MH is compared against the three classical types of D3, it stands out as having the most impact overall at population level (Figure 1). Notably, amelogenesis imperfecta can be quite devastating for the families concerned, as often all teeth are severely malformed leading to extreme risk for poor appearance, toothache, caries, and failed dental restorations (Parekh et al., 2014; Sneller et al., 2014). Yet, because of its rarity, this genetic disorder poses a comparatively small burden for society (Table 1). Likewise, in developed countries at least, fluorosis and (true) enamel hypoplasia are generally less prevalent than MH and don't carry as much risk for caries and toothache. MH-related decay may also be wrongly attributed to hypoplasia due to ignorance that the latter term describes enamel missing before eruption and not enamel loss that has occurred post-eruptively^7^. With such high burdens on individuals and society, it is quite remarkable that MH is not better recognised and understood. Although its key features were reported decades ago (Hurme, 1949; Suckling et al., 1976, 1987; Koch et al., 1987), MH has only come to prominence in the past 15 years and, even then, awareness resides mainly with specialists in children's dentistry. A likely reason is that, due to inadequate education about the diagnostic differentiators noted above, MH is widely misdiagnosed as regular caries (particularly in high-caries-risk situations). Consequently, MH remains a largely silent public health problem and its considerable socioeconomic costs may be misassigned to caries. With few researchers currently engaged on this topic, MH offers numerous opportunities for worthy investigation.

## A multi-faceted research attack is needed for molar hypomineralisation

As MH is a complex problem from both aetiological and healthcare perspectives, a multi-faceted research approach should prove beneficial. Research needs can be divided into two broad areas, namely better dental care for now and avenues toward medical prevention in the future. Within each area there are opportunities for several disciplines including public health, dentistry, medicine, and basic science. To understand the idiosyncrasies of MH (Table 1), investigations are needed at population, individual case and tooth levels. For example, respective enigmas include reasons behind (1) the large variations in MH prevalence reported from studies of nominally similar populations;^8^ (2) the asymmetric attack pattern at mouth level, whereby anywhere from one to all-four 6-year molars can be affected; and (3) the biophysical properties of demarcated opacities, whose discoloured enamel varies from having chalk-like softness to near-normal hardness. Given their past victories informing a variety of clinical issues (see Box 1), enamel researchers are well-positioned to advance the understanding of demarcated opacities and MH. Much potential exists for established enamel-research methodologies to be deployed on MH, both de novo and in comparison with the better understood classical D3s (Table 1).

## Scientific drilling into demarcated opacities

Being the defining pathology of MH, demarcated enamel opacities occupy centre stage not only clinically but also as a potential goldmine for aetiology. By 1949, it was recognised that these sharply-bordered opacities are clinicopathologically distinct, differing from the so-called “diffuse opacities” (the signature lesion of dental fluorosis) and the early stages of caries termed “white-spot lesions” (Hurme, 1949). Thirty years later in New Zealand, animal studies provided experimental verification and established that demarcated opacities arise during the hardening (maturation) stage of enamel formation, unlike enamel hypoplasias which occur in the preceeding secretory stage. A hypothesised link with infectious disease was supported in an infantile sheep model, but parallel epidemiology on children failed to reveal any concrete medical associations^9^. Perplexingly, this causal enigma persists today despite more than 30 aetiological studies of childhood populations around the globe (Suckling et al., 1987; Silva et al., 2016). Much potential exists for enamel researchers to refine these and other past findings, for example by using modern understanding of enamel maturation to narrow down the developmental period when immature enamel is at risk of MH (Suckling, 1980; Smith, 1998; Lacruz et al., 2011; Robinson, 2014).

Recently, a possible breakthrough has come from proteomic investigation of “chalky” demarcated opacities (Mangum et al., 2010). Such opacities, which are found in moderate and severe cases of MH, present clinically as being unusually soft and discoloured in shades of cream, yellow, and brown. By comparing normal enamel with chalky opacities bearing intact and broken surfaces, a unique correlation was found between chalky enamel and albumin—a protein normally found in blood, tissue fluid, and saliva. This observation, which we have since verified using additional methodology and specimens (VP, JM, MH; unpublished data), resonates with an earlier speculation that albumin may have an inhibitory effect on enamel mineralisation during the development of so-called “white spot hypoplasias” (Robinson et al., 1992). However support at that time was controversial, coming from post-mortem animal specimens and being contradicted by albumin-transcript and human-derived data (Couwenhoven et al., 1989; Mangum et al., 2010). Moreover, another group's proteomic investigation produced somewhat different results by revealing albumin in both normal enamel and demarcated opacities, along with other proteins usually associated with saliva (Farah et al., 2010). Clearly, further work is required to verify the hypothesised developmental link between albumin and chalky enamel and to rule out alternative explanations such as post-eruptive contamination with blood or saliva. A second finding from proteomic profiling was nearly complete absence of the principal enamel protein, amelogenin, from chalky opacities which in turn led to MH being categorised as a hypocalcification defect by analogy to the various phenotypes of amelogenesis imperfecta. This result argued against abnormal retention of amelogenin being primarily responsible for chalky enamel (as holds for some types of amelogenesis imperfecta and fluorosis), and instead added support to the albumin hypothesis (Mangum et al., 2010). Provocatively, these proteomic findings suggest that other aetiological clues might remain preserved within chalky enamel. Complementing the proteomic approach, murine toxicology models have been used to investigate whether antibiotics and environmental toxins cause MH (Laisi et al., 2009; Jedeon et al., 2013; see also Kirkham et al., 2017). Although various effects on enamel development were reported, it is uncertain how closely these animal models emulate the human situation in terms of opacity characteristics and typical childhood exposures. Regardless, much potential exists for enamel researchers to pursue such avenues and develop rigorous animal models for demarcated opacities and MH (Suckling et al., 1983; see also Kirkham et al., 2017).

Considering past gains (see Box 1), enamel researchers could sensibly tackle many other questions in this area besides those related to pathomechanism and causation. For example, of particular clinical relevance are correlations between physicochemical properties and opacity appearance, and also the notion of using allied chemical and molecular information to assist diagnosis and treatment (Mahoney et al., 2004; Mangum et al., 2010; Natarajan et al., 2015). Currently dentists have little other than hard-won experience to go on when deciding whether a moderate-grade opacity might be preserved using remineralisation strategies, or will instead break down under chewing forces and progress rapidly to severe decay. The role of genetics in MH risk is another area yet to be clarified, including by use of twin studies. It is noteworthy in this regard that, although not primarily genetic in origin (unlike amelogenesis imperfecta), fluorosis and caries-risk both appear to have underlying genetic contributions (Everett et al., 2002; Shaffer et al., 2011; Vieira and Kup, 2016).

To maximise research traction, it will be important to amalgamate clinical and scientific viewpoints on demarcated opacities, and to develop cross-compatible research frameworks and terminologies^10^. And in deciding how best to move forward, it will also be valuable to reevaluate historic data which in many cases remains pertinent to what is today described as MH, despite diagnostic and terminological differences^11^. It follows from such ambitions that further scientific drilling into the medical origins, clinical properties and treatment of demarcated opacities will best be done collaboratively by cross-disciplinary teams. Moreover, while as in the past there remains an important role for individual contributions, it seems that an expansive medico-dental research problem of this nature will benefit from a networked research approach whereby various participatory groups are brought together into a more powerful whole.

## The need for a cross-sector, translational approach to molar hypomineralisation

Simply put, “the MH problem” can be seen to comprise three aspects—(1) the health issues surrounding MH, (2) the under-recognition of this silent health problem, and (3) the paucity of research-based understanding of MH. If MH is to be treated better in the near-term and ultimately prevented, improvements to research and awareness are required. Key topics for awareness-building include the socioeconomic gravity of MH, its common misdiagnosis as regular dental caries, and the worthiness of preventive research. These messages need to be delivered across the sector, thereby informing the at-risk public and politicians serving them, the healthcare providers and their educators, the supporting industry, and the medico-dental research community. With so many unknowns and so much at stake, researchers need encouragement to address not just the amount of research they do, but also its quality as seen through translational eyes. It follows that a cross-sector, translational network approach to the MH problem stands to benefit research, education, and public-health policy. For such an approach to work, diverse research capabilities must be attracted to this nascent field, particularly from basic science, paediatric medicine, dentistry, public health, and allied industry. Many strategic advantages would accrue if such a network was to have global reach, recognising for example that, as for childhood caries, the MH problem will have socio-geographic variations. Likewise, educational benefits will flow from consistent messaging, and a “networked research army” should act as a high-capability magnet for the requisite major resourcing. With these ideas in mind, a translational network was recently piloted across Australia and New Zealand. Using cross-sector inputs, this initiative (The D3 Group) has launched a public awareness campaign^12^, a children's storybook^13^, and a comprehensive online-education resource serving diverse target audiences (i.e., affected children and families, public health sector and politicians, medical and dental practitioners, researchers)^14^. A key element of these initial steps was the drafting of a translational lexicon that embraces diverse needs including public-friendliness (“chalky teeth”), ease of speech (“D3s”), and a public-health focus on decay-prone molars whilst respecting the clinicopathological importance that any tooth can be affected by demarcated opacities (“Molar Hypomineralisation”). Realising the enamel-science community is small and scattered, it is hoped that further internationalisation of The D3 Group network will provide an empowering vehicle that attracts more enamel researchers (amongst many others) to join the worthy fight against MH.

## Author contributions

MH conceived, designed and drafted the article and figures. VP and JM helped conceive the D3 comparisons and Past Victories sections, respectively, and added other intellectual content. As originators of “D3 Dad's Army” (i.e., senior practitioners contributing hands-on to basic research in the Hubbard group), GN and RH have made numerous intellectual contributions to the perspective outlined here. All authors refined and reviewed the final manuscript.

### Conflict of interest statement

The authors declare that the research was conducted in the absence of any commercial or financial relationships that could be construed as a potential conflict of interest.

## Footnotes

^1^www.thed3group.org/what-is-d3.html

^2^http://www.chalkyteeth.org

^3^www.thed3group.org/prevalence.html

^4^www.thed3group.org/the-basics-2.html

^5^www.thed3group.org/health-risks.html

^6^www.thed3group.org/economic-cost.html

^7^www.thed3group.org/the-basics.html

^8^www.thed3group.org/prevalence.html

^9^www.thed3group.org/grace-suckling.html

^10^www.thed3group.org/the-basics.html

^11^www.thed3group.org/grace-suckling.html

^13^www.chalkyteeth.org

^15^www.thed3group.org/sam-has-molar-hypomin.html

^17^www.thed3group.org

## Abbreviations

AI: amelogenesis imperfecta
D3: developmental dental defect
MH: Molar Hypomineralisation.
